# Measurement invariance of the moral vitalism scale across 28 cultural groups

**DOI:** 10.1371/journal.pone.0233989

**Published:** 2020-06-09

**Authors:** Maksim Rudnev, Christin-Melanie Vauclair, Samira Aminihajibashi, Maja Becker, Michal Bilewicz, José Luis Castellanos Guevara, Emma Collier-Baker, Carla Crespo, Paul Eastwick, Ronald Fischer, Malte Friese, Angel Gomez, Valeschka Guerra, Katja Hanke, Nic Hooper, Li-li Huang, Minoru Karasawa, Peter Kuppens, Steve Loughnan, Müjde Peker, Cesar Pelay, Afroditi Pina, Marianna Sachkova, Tamar Saguy, Junqi Shi, Mia Silfver-Kuhalampi, Florencia Sortheix, William Swann, Jennifer (Yuk-Yue) Tong, Victoria Wai-lan Yeung, Brock Bastian

**Affiliations:** 1 National Research University Higher School of Economics, Moscow, Russia; 2 Instituto Universitário de Lisboa (ISCTE-IUL), CIS-IUL, Lisboa, Portugal; 3 Department of Psychology, University of Oslo, Oslo, Norway; 4 CLLE, Université de Toulouse, Toulouse, France; 5 University of Warsaw, Warsaw, Poland; 6 ConSol, Mexico, Mexico; 7 University of Queensland, Brisbane, Australia; 8 CICPSI, Faculty of Psychology, University of Lisbon, Lisbon, Portugal; 9 University of California, Davis, California, United States of America; 10 Victoria University of Wellington & Instituto D’Or de Pesquisa e Ensino, Wellington, New Zealand; 11 Saarland University, Saarbrucken, Germany; 12 Universidad Nacional de Educación a Distancia, Madrid, Spain; 13 Universidade Federal do Espírito Santo, Espírito Santo, Brazil; 14 University of Applied Management Studies, Mannheim, Germany; 15 University of the West of England, Bristol, United Kingdom; 16 National Taiwan University, Taipei, Taiwan; 17 Nagoya University, Nagoya, Japan; 18 KULeuven-University of Leuven, Leuven, Belgium; 19 University of Edinburgh, Edinburgh, Scotland; 20 MEF University, Istanbul, Turkey; 21 Universidad Central de Venezuela, Caracas, Venezuela; 22 University of Kent, Canterbury, United Kingdom; 23 Russian Presidential Academy of National Economy and Public Administration, Moscow, Russia; 24 Interdisciplinary Center (IDC) Herzliya, Herzliya, Israel; 25 Zhejiang University, Hangzhou, China; 26 University of Helsinki, Helsinki, Finland; 27 University of Texas Austin, Austin, Texas, United States of America; 28 Singapore Management University, Singapore; 29 Lingnan University, Hong Kong, Hong Kong; 30 University of Melbourne, Melbourne, Australia; Saint Peter’s University, UNITED STATES

## Abstract

Moral vitalism refers to a tendency to view good and evil as actual forces that can influence people and events. The Moral Vitalism Scale had been designed to assess moral vitalism in a brief survey form. Previous studies established the reliability and validity of the scale in US-American and Australian samples. In this study, the cross-cultural comparability of the scale was tested across 28 different cultural groups worldwide through measurement invariance tests. A series of exact invariance tests marginally supported partial *metric* invariance, however, an approximate invariance approach provided evidence of partial *scalar* invariance for a 5-item measure. The established level of measurement invariance allows for comparisons of latent means across cultures. We conclude that the brief measure of moral vitalism is invariant across 28 cultures and can be used to estimate levels of moral vitalism with the same precision across very different cultural settings.

## Introduction

Moral vitalism is a concept that captures how lay people think about and explain morally relevant actions and events in the world [1–2]. It is a lay theory that embraces the dual beliefs that forces of good and evil (a) actually exist and (b) may cause moral and immoral events to occur. Moral vitalism acts as a lay theory or heuristic for navigating the complex world of moral judgement and behavior and is attractive because it provides a convenient explanation for why good and bad things happen, as well as what makes people good or bad (cf. [3–5]). In order to measure moral vitalism, Bastian and colleagues [1] developed the Moral Vitalism Scale (MVS). The MVS had a high predictive validity, showing that those who endorsed moral vitalistic beliefs tend to worry about being possessed by evil, are more sensitive to being contaminated through direct or indirect contact with evil people due to the possibility of contagion, and are more concerned about maintaining their own mental purity. The measure also demonstrated high reliability across six studies. Based on these findings, it would appear that moral vitalists view immoral essences—the forces of evil—as having the capacity to “infect” and corrupt people’s minds and bodies, either through physical contact or mental content alone. The current study aims to investigate the degree to which the MVS has similar measurement characteristics across different cultures, that is, whether its scores can be used in cross-cultural research to examine relations of moral vitalism with other constructs and to test mean differences in vitalistic beliefs across cultures.

### Moral vitalism

The construct of moral vitalism provides an important avenue through which to examine the role of spiritual beliefs in moral reasoning and judgement. It goes beyond prior work focused on how a belief in God may shape thinking and behavior (e.g., [6]) or work focusing on concerns over sanctity or purity (e.g., [7]), by examining a general lay theory about spiritual forces that is not limited to specific religious commitments or political beliefs. Moral vitalism provides people with a convenient explanation for why good and bad things happen, as well as what makes people good or bad. Like other lay theories, moral vitalism may often be largely implicit and poorly articulated.

By focusing on how people explain their moral worlds, moral vitalism provides an account of moral cognition which diverges from the view that morality arises from the need to protect persons, groups, or norms (such as moral foundations theory, see [7]). While it shares some similarities with accounts that emphasize the role of sense making in the context of harm (such as the notion of dyadic completion: [8]), it goes beyond such accounts by suggesting that in efforts to understand their worlds, people often rely on beliefs that have explanatory power. Beyond completing a moral dyad of victim and perpetrator, moral vitalism serves to explain why there are victims and perpetrators in the first place. Yet, in contrast to theories focusing on the moral character of agents (e.g., [9]), moral vitalists see moral action as in part determined by forces that are independent of people, but which can possess and influence them (see [1]). Supportive of this, moral vitalism appears to be relatively distinct from a similar construct focusing on pure good and evil published by [10], but which focused on purely good or evil people, as opposed to purely good or evil forces (see [1]).

In short, moral vitalism provides a filler explanation, or a placeholder concept, for why morally relevant events occur. As an approach to understanding moral cognition, it suggests that morality may be as much characterized by a set of beliefs about the nature of the world as it is by the basis on which people make moral judgements.

As a formal theory, moral vitalism reflects a basic form of cognition (belief) that is likely universal and probably arose as an explanation for life threatening events, such as disease in contexts where other (i.e., more scientific) explanations were not available (see [11]). From this perspective, moral vitalism is perhaps most closely aligned with the purity dimension of moral foundations theory, given a similar emphasis on concerns regarding purity and contagion, and links to concerns over biological disease ([7, 12]). As such, moral vitalism is likely to be evident across a range of cultures, and yet it is also likely to be reinforced within particular cultural contexts.

The theory and measurement of moral vitalism provides exciting and novel avenues for cross-cultural research. For example, [11] showed in a cross-national study that historically higher levels of pathogen prevalence were positively associated with the endorsement of moral vitalistic beliefs. Other social ecological factors could also be explored, such as the prevalence and frequency of natural disasters, because they may equally be related to the development of moral vitalism as a cultural meaning-making belief system. Given that moral vitalism takes account of the role of spiritual belief within the moral domain by focusing on core underlying assumptions, rather than specific tenets of religious, cultural or political beliefs, it opens up new avenues for comparison across culturally diverse populations, and across varying ideological and religious commitments. A cross-nationally reliable scale on moral vitalism is able to provide novel insights into the factors that stimulated the development and preservation of such beliefs.

## Method

### The scale

The current version of the MVS features five items assessing the belief in real, agentic forces of good and evil (e.g., “There are underlying forces of good and evil in this world”) on a 6-point Likert scale ranging from 1 = strongly disagree to 6 = strongly agree [1]. The item wordings are presented in Table 1. The scale was translated from English into the respective languages of countries (see Table 2) by bilinguals. Accuracy of the translation was verified through back-translations or a committee approach. S1 File include translations of the MVS into these languages.

**Table 1:** Descriptive statistics of the five items comprising the moral vitalism scale.

Item labels	Item wording	Pooled sample means	Standard deviation	% missing
**existence**	There are underlying forces of good and evil in this world	3.76	(1.66)	.35
**responsible**	Either the forces of good or the forces of evil are responsible for most of the events in the world today	3.15	(1.62)	.26
**motivates**	The forces of good and evil often motivate human behavior	3.62	(1.66)	.38
**awareness**	People need to be aware of the good and evil that are in this world today	4.57	(1.37)	.42
**natural**	Good and evil are aspects of the natural world	3.87	(1.55)	.29

**Table 2:** Sample characteristics and the latent means of moral vitalism estimated by the partial approximate scalar invariance model (sorted by latent mean).

	% female*	Mean age*	Sample size	Moral vitalism latent mean	Posterior standard deviation	Language	City
Indonesia	81.0	20.0	100	.32	.37	Indonesian	Jakarta
USA	48.0	18.8	100	.16	.43	English	College Station, TX
Turkey	87.2	20.8	110	.04	.39	Turkish	Istanbul
Japan	13.1	19.7	154	0	0	Japanese	Nagoya
Northern Cyprus	43.8	21.3	80	-.34	.42	Turkish	Gezelyurt
Hong Kong	78.5	21.3	79	-.42	.39	English	Tuen Mun
Singapore	64.0	21.3	86	-.48	.41	English	Singapore
China	61.3	20.3	119	-.55	.37	Chinese	Guangzhou
Taiwan	51.0	19.6	104	-.63	.40	Chinese	Taipei
Mexico	73.0	25.4	100	-1.63	.57	Spanish	Cancún
UK	77.8	20.4	54	-1.88	.68	English	Edinburgh
New Zealand	73.9	19.6	142	-1.88	.52	English	Wellington
Venezuela	59.6	20.8	104	-2.22	.64	Spanish	Caracas
Israel	73.4	22.9	140	-2.35	.57	Hebrew	several
France	63.4	20.9	71	-2.41	.72	French	Toulouse
Russia	64.7	20.2	85	-2.47	.68	Russian	Moscow
Australia	71.1	20.9	87	-2.50	.71	English	Brisbane
Portugal	89.6	21.0	193	-2.62	.57	Portuguese	Porto
Poland	47.7	22.8	107	-2.82	.72	Polish	Warsaw
Brazil	52.3	22.6	111	-3.14	.75	Brazilian Portuguese	Vitoria
Greece	90.5	23.1	101	-3.22	.80	Greek	several
Belgium	85.7	18.6	160	-3.26	.71	Dutch	Leuven
Norway	49.4	23.4	78	-3.36	.81	Norwegian	Oslo
Spain	19.5	36.0	200	-3.88	.82	Spanish	several
Switzerland	77.1	23.9	118	-4.05	.86	German	Basel
Austria	90.9	24.7	56	-4.13	.93	German	Salzburg
Germany	72.3	23.3	103	-5.06	1.03	German	Bremen
Finland	77.7	27.8	187	-5.09	1.23	Finnish	Helsinki
***Total***	*64*.*4*	*22*.*6*	*3129*				

* 26 respondents did not indicate their gender and 34 respondents did not indicate their age.

### Data

A total of 3,202 undergraduate university students residing in 28 countries participated in this study. Ethical approval for this study was obtained by the last author from the University of Melbourne’s Behavioural and Social Sciences Ethical Review Committee, project no. 2009001486. Informed consent was obtained in line with the requirements of ethical approval. All other samples in this study were collected in line with relevant ethical protocols and informed consent procedures for each country.

The participants and cultures were sampled on a convenience basis. The central team reached researchers through professional networks and asked them to collect a sample of 80–100 respondents. Participants responded to a larger questionnaire that included the MVS. Respondents who took part in the study received non-monetary incentive such as course credits.

The average age of the total sample was 22.6 years (SD = 6.3), ANOVA test showed significant differences in age across samples. In Spain, the average age was substantially higher as the participants were students of an open university, which attracts more mature attendance. Gender composition was also significantly different across samples as indicated by a significant χ^2^ test. Overall 65% of all participants were female (see Table 2). The data contained less than one percent of cases with missing values, these were treated with full information maximum likelihood in frequentist models, whereas Bayesian models implicitly incorporated missing values.

### Analytical approach

The analysis followed three major steps. At first, we identified a pool of the items that showed similar factor structure across cultural groups. Second, we ran a series of full and partial exact invariance tests. Third, an approximate invariance approach was applied to adjust the model to the population more closely. At all stages we applied confirmatory factor analysis models using R package “lavaan” [13] and Mplus version 7.3 software [14]. All the codes with details of the analyses and the replication data can be found in the S1 File.

A conventional way to assess measurement invariance is to run a series of multiple groups confirmatory analyses [15]. First, a configural model is fitted to the data. Configural model does not constrain factor loadings or item intercepts. Second, a metric invariance model is fit to the data, which is similar to the configural model, but the factor loadings are constrained to be equal across groups. And finally, a scalar invariance model is fitted, which constrains both factor loadings and item intercepts. After fitting these three models to the data, they can be compared using likelihood ratio χ^2^ test. However, χ^2^ difference test was shown to be overly conservative with larger sample sizes [16]. Thus, another set of criteria was suggested: differences in comparative fit index (CFI) larger than .01 and difference in RMSEA larger than .015 as an indication of substantial differences between models [17]. If a configural model has a substantially better fit than a metric invariance model, it is preferred over the metric invariance model. Likewise, if a metric invariance model has a substantially better fit than a scalar invariance model, the former is preferred over the latter.

An important prerequisite to this sequence of model testing is a well-fitting configural model. As a criterion of an acceptable fit for a factor model we used CFI/TLI > .90, RMSEA < .08; SRMR < .08 [18]; χ^2^ was ignored in this analysis due to a large (over 3000) sample. The model was identified using a marker variable approach (see [19]). After a preliminary analysis, we decided to use item “existence” as a marker, because it showed highly invariant parameters across groups when using different model specifications. Switching of a marker variable to the other items did not affect substantive results.

At the second stage of analysis we tested several partial invariance models. Byrne, Shavelson, and Muthén [20] proposed the idea of partial invariance, which claims that some of the loadings/intercepts are allowed to vary without creating a substantial bias. They suggested that it is sufficient to have two invariant loadings for a partial metric invariance and two invariant intercepts for a partial scalar invariance. However, the consecutive tests of partial invariance might lead to an inductive solution which brings a danger of overfitting, that is, a possibility that the final model may not replicate with different data. For this reason, we were especially careful in handling model modifications which were not expected by the theory.

The third stage of analysis was based on Bayesian statistics with informative priors. The prior between-group difference in factor loadings and/or item intercepts was set to zero (no differences) and its variance was set to a conventionally low value. The between-group variance in factor loadings and intercepts of the size .01 was considered negligibly small for most substantive conclusions (it defines the 95% confidence interval of absolute differences in parameters of .20, see also [21]). Between-group variance in parameters of .10 was considered large and deviating from the invariance (corresponds to ±.63 difference on a standardized scale).

First, we tested an approximate configural model, in which the factor loadings and item intercepts were allowed to vary with a prior between-group variance of .10. Given a well-fitting configural model, we then set the prior between-group variance to .01 for loadings (approximate metric invariance) and then for both loadings and intercepts (approximate scalar invariance). In order to evaluate the model fit to the data, two key statistics were examined: 95% confidence interval for the difference between the observed and the replicated χ^2^ values (if it includes zero, it indicates acceptable fit), and related standardized index of posterior predictive p-value (PPP, which should be higher than .05 with a perfect fit indicated by value of .50). As long as the sample sizes were relatively small, we expected convergence issues. To ensure convergence the models were run using 4 chains and a minimum of 30,000 iterations. All the models revealed stable solutions indicated by potential scale reduction factor (R-hat), which was smaller than 1.1 for every parameter. Scanning of trace plots did not reveal issues with convergence as well. Visual examination of autocorrelation plots detected no substantial problems with the parameter estimation.

## Results

### Identifying the pool of items

The MVS initially included eight items: three reverse-coded items and five non-reversed items. Non-reversed items are listed in Table 1; reversed items were following: “Good and evil are human constructions”, “Things happen and sometimes they have good or evil consequences, but there is nothing that is truly good or truly evil”, “There is nothing that is really good or really evil in this world, it’s all a matter of perspective”. A preliminary analysis on the pooled sample demonstrated acceptable fit of a two-factor model: one factor loaded on all the reverse-coded items and the other factor loaded on the rest of items [CFI = .964, RMSEA = .069, SRMR = .045, χ^2^(df = 19) = 299]. However, applying this model in the multiple group settings was challenging, as in many groups it resulted in a non-positive definite matrix, which signaled potential problems with the model and the data (such as high multicollinearity combined with small samples). Moreover, running the two-factor model separately in each group resulted in various model problems across groups.

We concluded that configural invariance across groups could not be detected with the eight items. Therefore, we dropped the reverse-coded items and examined measurement invariance for a one-factor model with the five straight-coded items only.

### Exact measurement invariance tests

**Fig 1:**
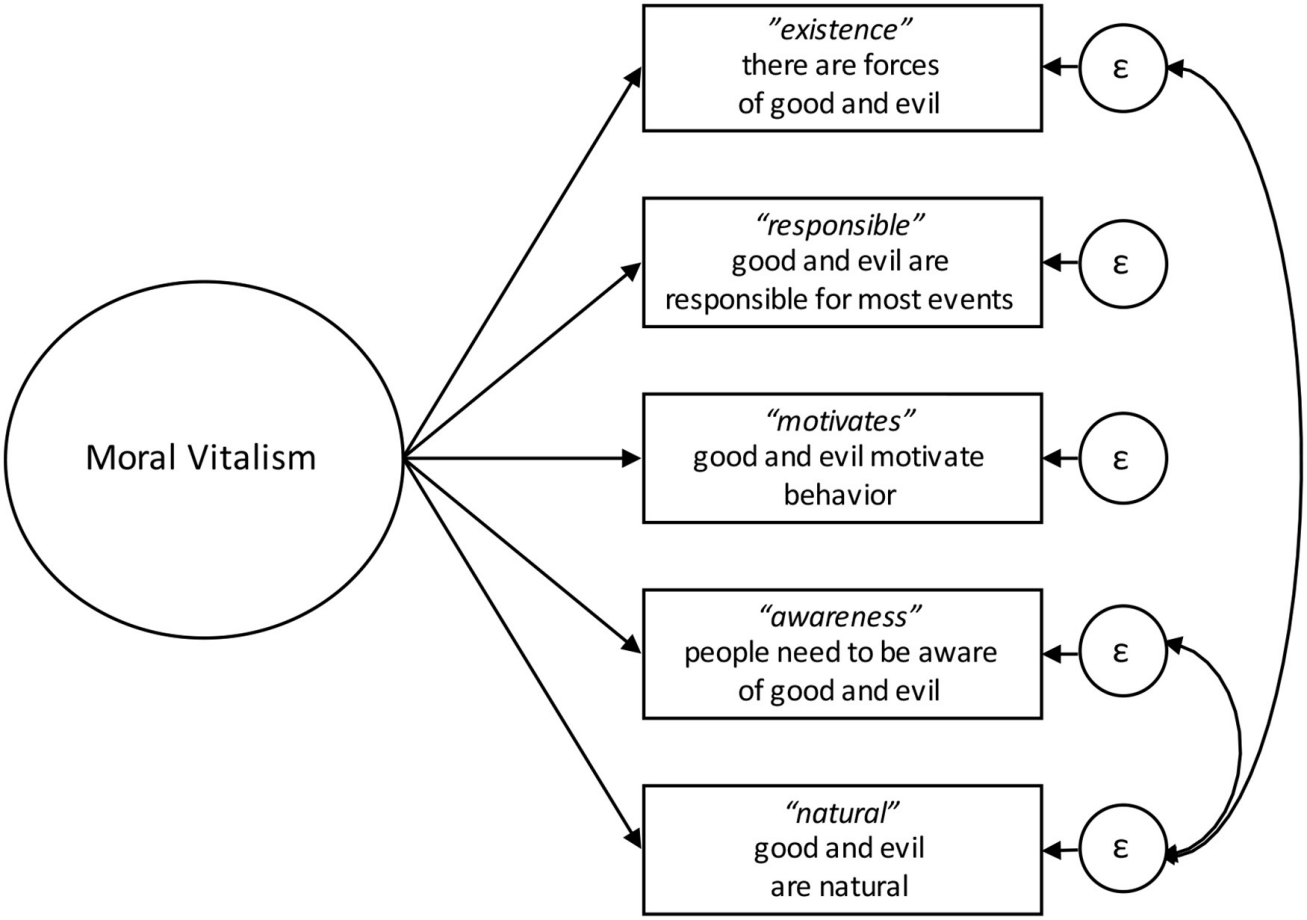
Measurement model of moral vitalism scale.

A simple multiple-group factor model with a single factor at the pooled sample showed unacceptable fit as indicated by a high value of RMSEA (.098; the other fit measures were within the recommended range, CFI = .971, TLI = .942). Modification indices suggested adding a covariance between items “awareness” and “natural” which improved the model and yielded a marginally acceptable value of .078 for RMSEA. At the next step, the modification indices suggested to add a covariance between residuals of the items “natural” and “existence” which makes sense, because both items claim the existence of good and evil forces in a natural world. The resulting model (Fig 1) had appropriate fit in a multiple-group setting (see model 1 in Table 3), as well as it fitted to the data from each country separately–*p*-values of χ^2^ tests of model fit were above .01 in every group. Therefore, this model was further used in the invariance testing as a configural model. The alternative model with two factors, one including “existence”, “natural”, and “responsible”, and the other including “motivates” and “awareness” showed acceptable fit on the pooled sample but did not converge in multiple-group analysis (configural model).

**Table 3:** Exact measurement invariance tests of the single-factor model of moral vitalism.

	Model	CFI	ΔCFI	RMSEA	ΔRMSEA	SRMR	ΔSRMR	χ^2^	Df
***Full measurement invariance with 5 items and covariance between “awareness” and “natural” and between “existence” and “natural”***									
1	Configural	.992		.066		.023		124.6	84
2	Metric	.947	.045	.113	.047	.101	.078	465.7	192
3	Scalar	.810	.137	.172	.059	.150	.048	1286.5	300
4	Partial metric, loadings of “existence” and “awareness” are allowed to vary across groups	.982	.011	.079	.013	.056	.033	233.7	138
5	Partial scalar, loadings and intercepts of “existence” and “awareness” are allowed to vary across groups	.934	.047	.126	.047	.082	.026	532.6	192
6	Partial metric, loadings of “existence”, “awareness” and “natural” are allowed to vary across groups	.987	.005	.074	.008	.036	.013	178.3	111
7	Partial scalar, loadings and intercepts of “existence”, “awareness” and “natural” are allowed to vary across groups	.973	.014	.096	.022	.044	.008	279.7	138

Table 3 lists fit indices from a series of the exact measurement invariance tests with multiple group confirmatory factor analyses. Constraining factor loadings across groups to equality, that is, testing the metric invariance, decreased CFI by .045 and increased RMSEA by .034, both values are much greater than the recommended ones. Therefore, we had to select configural invariance model over metric invariance and concluded that there is no full metric invariance.

Next, we tested partially invariant models. We examined the factor loadings in each group from the configural model in which they were estimated independently in every group. This information as well as modification indices indicated that factor loadings of items “existence” and “awareness” had the largest differences across groups, so we relaxed these equality constraints. Comparing the model fit of the partial metric invariance model (model 4) to the fit of the configural model revealed a decrease in CFI of .011 and increase in RMSEA of .013, which almost fell in the recommended range. So, we were able to conditionally accept this partial metric invariance model. However, when we tested the partial scalar invariance model, the decrease in CFI and increase in RMSEA were very large (model 5), thus, it was rejected. We also tried a minimal partial metric invariance model, releasing equality constraints on loadings of “existence”, “nature”, and “awareness” items, which expectedly showed an acceptable decrease in the model fit (model 6) compared to configural invariance model. This model was then used to test for the partial scalar invariance (model 7), a decrease in CFI was .014, close to the suggested threshold, but increase in RMSEA was .022, substantially larger than the recommended threshold of .015, and the absolute value of RMSEA of .096 pointed to the model’s overall poor fit to the data.

The resulting partial metric invariance model is prone to criticism, because, first, the RMSEA statistic, although technically being under the recommended threshold of .08, had an upper confidence bound which was beyond this threshold. Moreover, these models had a very few degrees of freedom, which may imply that the models could have been overfitted, that is, be applicable only to the current sample. Given the nature of the convenience sample, the problem of overfitting is especially important issue, so the minimally fitting model might not be the population model. Due to these concerns, we switched to the approximate measurement invariance approach [21–22], which in a way avoids the problem of overfitting by allowing some across-group variance in item intercepts and factor loadings.

### Approximate invariance approach

Table 4 lists the model fit statistics of approximate invariance testing. The approximate configural model fits the data very well, showing PPP of .164 and a confidence interval of χ^2^ including zero. The metric model showed a similarly good fit with PPP = .060. The scalar invariance model showed a deterioration in fit with a PPP dropping below the recommended threshold of .05 to essentially zero, and BIC increasing compared to the metric and configural models. Increasing prior variance of the intercepts of the items “natural”, “awareness”, and “responsible” resulted in a partial approximate scalar invariance model that fitted the data sufficiently well: PPP of .061 was in the recommended range, χ^2^ confidence interval included zero and the increase in BIC was relatively small. We consider this model final and conclude that the measurement model of moral vitalism with five items and two residual covariances (of the items “natural” with “awareness” and “existence”) is approximately and partially invariant at the scalar level, which implies that the means and regression/correlation coefficients can be compared across all 28 cultural groups.

**Table 4:** Model fit indices of approximate invariance tests.

	PPP	χ^2^ 95 CI LB	χ^2^ 95 CI UB	BIC	pD
Good fit criteria [23]	>.05	Includes zero		Small	
1 Configural (prior between-group variance of loadings and intercepts is .10)	0.165	-46.5	137.6	39368	380.4
2 Metric (prior between-group variance of loadings is .01, but for intercepts it is .10)	0.074	-25.9	162.6	39412	385.8
3 Scalar invariance (prior between-group variance of loadings and intercepts is .01)	0.001	56.4	247.2	39533	350.8
4 Partial scalar (prior between-group variance of loadings and intercepts is .01, intercept of “natural”, “awareness” and “responsible” variance set to .10)	0.066	-20.2	166.0	39419	382.9

95 CI LB and 96 CI UB stand for 95% confidence interval, lower bound and upper bound, respectively. pD is estimated number of parameters.

The latent means estimated by the partial approximate scalar invariance model are listed in Table 2. The latent mean had to be fixed to zero in one of the groups, so we randomly chose Japan. The other means represent differences in moral vitalism compared to Japan. Expectedly, Western European countries occupied the lower ranks, while Asian cultures as well as USA and Cyprus scored the highest on moral vitalism. Previous analyses have shown that country-level the moral vitalism score correlated robustly with pathogen prevalence, therefore, underscoring the cross-cultural validity of the scale [11]. Interestingly, the lower levels of moral vitalism tended to coincide with the larger confidence interval of the estimate.

It is noteworthy that the latent means estimated by different models were highly similar. For example, the latent means from the exact scalar invariance model (model 3 in Table 3 which showed poor fit) correlated with the means estimated by the approximate partial scalar invariance model (model 4 in Table 4) at .99.

## Discussion and conclusion

In this paper we aimed at testing a novel scale of moral vitalism across 28 cultural groups. A model with five items, a single factor and covariance of residuals for three items showed an acceptable fit. The multiple tests of measurement invariance indicated the lack of exact scalar as well as full metric invariance. The tests of approximate measurement invariance supported the presence of partial scalar invariance, where only intercepts of item “natural” were allowed to vary across groups. This conclusion allowed to correctly estimate latent means: Asian countries as well USA and Northern Cyprus tended to be high on moral vitalism, while Western Europeans are less prone to follow this belief.

The results of the analysis demonstrated that MVS can be used to correctly measure moral vitalism in a wide range of cultures. Moreover, the results suggest that the concept of moral vitalism has approximately the same meaning across these cultures, whereas in general the lay theories of good and evil are intertwined with specific religious and cultural beliefs.

The current study has several limitations. First, the convenience sampling of the student participants may have affected the results, which might be biased towards younger, female, and educated individuals, rather than reflecting the parameters of the general population. However, a systematic pattern of latent means found in the study demonstrated that the group differences do indeed reflect cultural differences in the moral vitalism beliefs. Moreover, the use of similar samples across national contexts helped to tackle cultural differences while holding other differences across samples constant.

Another limitation regards the exclusion of reversed-coded items at the preliminary analysis stage. It might have affected the results in a way that the final score and respective latent means of moral vitalism are likely to also include response tendencies, such as acquiescence and non-differentiation. It might not be problematic as [1] demonstrated that the measure had criterion and predictive validity regardless of the lack of balance in item wordings. However, future research should investigate this issue in depth.

Yet another potential limitation is related to the fact that we detected only a weak evidence of scalar invariance–that is, the initial model was appended with the two residual covariances and only two out of five item intercepts were found to be invariant across all the countries under study. Notwithstanding the differences in model fit, various models tested in the study estimated highly similar factor means which correlated across countries at .99 or higher. It indicates that the small differences in item intercepts and factor loadings did not substantively affect the latent means proving their reliability. It suggests that the poor fit of the exact scalar invariance model was due to some noise in the data unrelated to the problems of measurement invariance itself.

Despite these limitations, our results provided evidence of MVS ability to reliably measure moral vitalism in a wide range of cultures which allows comparison of latent means and correlations/regression coefficients across a wide range of cultures. Establishing the cross-cultural invariance of moral vitalism opens new lines of inquiry into cultural variability in naïve theories of spirituality and morality.

## Supporting information

S1 File (ZIP)
