# Supplementary material for: Measurement invariance of the moral vitalism scale across 28 cultural groups
Source: PLoS One. 2020 Jun 9;15(6):e0233989. doi: 10.1371/journal.pone.0233989 (PMC7282638; doi:10.1371/journal.pone.0233989)
Supplement: S1 File — (ZIP) [file pone.0233989.s001.zip › QuestionnaireTranslations.docx]

Supplementary Material for: Rudnev M., Vauclair C.-M… & Bastian B. Measurement invariance of the moral vitalism scale across 28 cultural groups

**Translations of the Moral Vitalism Scale**

[Source English 2](#_Toc38011496)

[Dutch (Belgium) 3](#_Toc38011497)

[Finnish 4](#_Toc38011498)

[French 5](#_Toc38011499)

[German 6](#_Toc38011500)

[Greek 7](#_Toc38011501)

[Hebrew 8](#_Toc38011502)

[Japanese 9](#_Toc38011503)

[Polish 10](#_Toc38011504)

[Portuguese (Brazilian) 11](#_Toc38011505)

[Portuguese (European) 12](#_Toc38011506)

[Russian 13](#_Toc38011507)

[Spanish (Spain) 14](#_Toc38011508)

[Spanish (Mexico) 15](#_Toc38011509)

[Turkish 16](#_Toc38011510)

Source English

| The following statements are about how you think about **good** and **evil**. There are **no right or wrong answers**. We are simply interested in how much you **agree** with each of the following statements. We are interested in your first responses, so please do not take too much time to think about each question. Please use the following scale to indicate how strongly you **agree** or **disagree** with each statement. |
| --- |

| **Strongly**  **disagree**  **1** | **Moderately**  **disagree**  **2** | **Slightly**  **disagree**  **3** | **Slightly**  **agree**  **4** | **Moderately**  **agree**  **5** | **Strongly**  **agree**  **6** |
| --- | --- | --- | --- | --- | --- |

| 1. There are underlying forces of good and evil in this world. | 1 | 2 | 3 | 4 | 5 | 6 |
| --- | --- | --- | --- | --- | --- | --- |
| 1. Either the forces of good or the forces of evil are responsible for most of the events in the world today. | 1 | 2 | 3 | 4 | 5 | 6 |
| 1. The forces of good and evil often motivate human behaviour. | 1 | 2 | 3 | 4 | 5 | 6 |
| 1. People need to be aware of the good and evil that are in this world today. | 1 | 2 | 3 | 4 | 5 | 6 |
| 1. Good and evil are aspects of the natural world. | 1 | 2 | 3 | 4 | 5 | 6 |

Dutch (Belgium)

| De volgende stellingen gaan over hoe jij denkt over goed en slecht. Er zijn geen juiste of foute antwoorden. We zijn gewoon geïnteresseerd in hoe sterk je het eens bent met de volgende stellingen. Gebruik de volgende schaal om aan te duiden hoe sterk je het eens of oneens bent met elke stelling. |
| --- |

| **Sterk oneens**  **1** | **2** | **3** | **4** | **5** | **Sterk eens**  **6** |
| --- | --- | --- | --- | --- | --- |

| 1. Er bestaan onderliggende krachten van goed en slecht in deze wereld. | 1 | 2 | 3 | 4 | 5 | 6 |
| --- | --- | --- | --- | --- | --- | --- |
| 1. De meeste gebeurtenissen in de wereld vandaag worden ofwel door de krachten van het goede of door de krachten van het slechte beïnvloed. | 1 | 2 | 3 | 4 | 5 | 6 |
| 1. De krachten van goed en slecht motiveren vaak menselijk gedrag. | 1 | 2 | 3 | 4 | 5 | 6 |
| 1. Mensen moeten zich bewust zijn van het goede en het kwade dat in deze wereld vandaag bestaat. | 1 | 2 | 3 | 4 | 5 | 6 |
| 1. Goed en slecht zijn aspecten van de natuurlijke wereld. | 1 | 2 | 3 | 4 | 5 | 6 |

Finnish

| Seuraavat väittämät käsittelevät sitä, miten ajattelet hyvästä ja pahasta. Ei ole oikeita tai vääriä vastauksia. Olemme yksinkertaisesti kiinnostuneita siitä, missä määrin olet samaa mieltä tai eri mieltä kunkin seuraavan väittämän kanssa. Olemme kiinnostuneita ensimmäisenä mieleen tulevista vastauksista, joten älä pohdi kysymyksiä liian kauan. Ole hyvä ja käytä seuraavaa asteikkoa merkitäksesi, kuinka vahvasti olet samaa tai eri mieltä kunkin väittämän kanssa. |
| --- |

| **Vahvasti eri mieltä**  **1** | **Jonkin verran eri mieltä**  **2** | **Hieman eri mieltä**  **3** | **Hieman samaa mieltä**  **4** | **Jonkin verran samaa mieltä**    **5** | **Vahvasti samaa mieltä**  **6** |
| --- | --- | --- | --- | --- | --- |

| 1. Tässä maailmassa on taustalla vaikuttavia hyvän ja pahan voimia. | 1 | 2 | 3 | 4 | 5 | 6 |
| --- | --- | --- | --- | --- | --- | --- |
| 1. Joko hyvän tai pahan voimat ovat vastuussa useimmista maailman nykyisistä tapahtumista. | 1 | 2 | 3 | 4 | 5 | 6 |
| 1. Hyvän ja pahan voimat motivoivat usein ihmisen käyttäytymistä. | 1 | 2 | 3 | 4 | 5 | 6 |
| 1. Ihmisten tulee olla tietoisia hyvästä ja pahasta, jotka ovat maailmassa nykypäivänä. | 1 | 2 | 3 | 4 | 5 | 6 |
| 1. Hyvä ja paha ovat luonnollisen maailman ilmiöitä. | 1 | 2 | 3 | 4 | 5 | 6 |

French

| Les énoncés suivants portent sur la façon dont vous pensez le bien et le mal. Il n’y a pas de bonnes ou de mauvaises réponses. Nous sommes simplement intéressés par votre degré d’accord avec chacun des énoncés suivants. Nous sommes intéressés par vos premières réponses, donc veuillez ne pas prendre trop de temps pour réfléchir à chacune des questions. S’il vous plaît, utilisez l’échelle suivante pour indiquer dans quelle mesure vous êtes en accord ou en désaccord avec chaque énoncé. |
| --- |

| **Fortement en désaccord**  **1** | **Modérément en désaccord**  **2** | **Légèrement en désaccord**  **3** | **Légèrement en accord**  **4** | **Modérément en accord**  **5** | **Fortement en accord**  **6** |
| --- | --- | --- | --- | --- | --- |

| 1. Il y a des forces sous-jacentes du bien et du mal dans ce monde. | 1 | 2 | 3 | 4 | 5 | 6 |
| --- | --- | --- | --- | --- | --- | --- |
| 1. Les forces du bien, ou les forces du mal, sont responsables de la plupart des évènements dans le monde aujourd’hui. | 1 | 2 | 3 | 4 | 5 | 6 |
| 1. Les forces du bien et du mal sous-tendent souvent le comportement humain. | 1 | 2 | 3 | 4 | 5 | 6 |
| 1. Les gens doivent être conscients du bien et du mal présents dans ce monde aujourd’hui. | 1 | 2 | 3 | 4 | 5 | 6 |
| 1. Le bien et le mal sont des aspects du monde naturel. | 1 | 2 | 3 | 4 | 5 | 6 |

German

| In den folgenden Aussagen geht es darum, wie Sie über **gut** und **böse** denken. Es gibt **keine richtigen oder falschen** **Antworten**. Wir sind einfach daran interessiert, inwieweit Sie den folgenden Aussagen **zustimmen**. Wir sind an Ihrer spontanen Antwort interessiert, aus diesem Grund bitten wir Sie nicht zu viel über Ihre Antwort nachzudenken. Bitte benutzen Sie folgende Skala, um anzugeben, wie sehr Sie den folgenden Aussagen **zustimmen** oder **nicht zustimmen**. |
| --- |

| **Stimme ganz und**  **gar nicht zu**  **1** | **Stimme**  **nicht zu**  **2** | **Stimme eher nicht zu**  **3** | **Stimme**  **eher zu**  **4** | **Stimme**  **zu**  **5** | **Stimme voll**  **und**  **ganz zu**  **6** |
| --- | --- | --- | --- | --- | --- |

| 1. Es gibt zugrunde liegende Mächte von Gut und Böse | 1 | 2 | 3 | 4 | 5 | 6 |
| --- | --- | --- | --- | --- | --- | --- |
| 1. Entweder die Mächte des Guten oder die Mächte des Bösen sind verantwortlich für die meisten Ereignisse dieser Welt heutzutage | 1 | 2 | 3 | 4 | 5 | 6 |
| 1. Die Mächte von Gut und Böse motivieren oft das Verhalten von Menschen. | 1 | 2 | 3 | 4 | 5 | 6 |
| 1. Heutzutage müssen Menschen sich dessen bewusst sein, dass es in dieser Welt Gut und Böse gibt. | 1 | 2 | 3 | 4 | 5 | 6 |
| 1. Gut und Böse sind Aspekte einer natürlichen Welt | 1 | 2 | 3 | 4 | 5 | 6 |

Greek

| Οι ακόλουθες δηλώσεις αφορούν στο πώς σκέφτεστε για το **καλό** και το **κακό**. **Δεν υπάρχουν σωστές ή λάθος απαντήσεις**. Ενδιαφερόμαστε απλώς για το πόσο **συμφωνείτε** με κάθε μία από τις ακόλουθες δηλώσεις. Μας ενδιαφέρει η πρώτη αντίδραση σας, οπότε μην πάρετε πάρα πολύ χρόνο για να σκεφτείτε την κάθε ερώτηση. Παρακαλούμε χρησιμοποιήστε την παρακάτω κλίμακα για να δείξετε πόσο έντονα **συμφωνείτε** ή **διαφωνείτε** με κάθε δήλωση. |
| --- |

| 1= Διαφωνώ απόλυτα | 2= Διαφωνώ μέτρια | 3= Διαφωνώ ελαφρώς | 4= Συμφωνώ ελαφρώς | 5= Συμφωνώ μέτρια | 6= Συμφωνώ απόλυτα |
| --- | --- | --- | --- | --- | --- |

| 1. Υπάρχουν ελλοχεύουσες (βαθύτερες) δυνάμεις του καλού και του κακού σ’ άυτον τον κόσμο | 1 | 2 | 3 | 4 | 5 | 6 |
| --- | --- | --- | --- | --- | --- | --- |
| 1. Είτε οι δυνάμεις του καλού, είτε οι δυνάμεις του κακού είναι υπεύθυνες για τα περισσότερα από τα γεγονότα στον κόσμο σήμερα. | 1 | 2 | 3 | 4 | 5 | 6 |
| 1. Οι δυνάμεις του καλού και του κακού συχνά παρακινούν την ανθρώπινη συμπεριφορά. | 1 | 2 | 3 | 4 | 5 | 6 |
| 1. Ο κόσμος πρέπει να έχει επίγνωση του καλού και του κακού που βρίσκονται σε αυτόν τον κόσμο σήμερα. | 1 | 2 | 3 | 4 | 5 | 6 |
| 1. Το καλό και το κακό ειναι πτυχές του φυσικού μας κόσμου | 1 | 2 | 3 | 4 | 5 | 6 |

Hebrew

| ההיגדים הבאים נוגעים לדרך בה את/ה תופס/ת **טוב מול רשע**. **אין תשובות נכונות או לא נכונות**. אנו מבקשים לדעת עד כמה  את/ה **מסכים/ה** עם ההיגדים הבאים. אנו מתעניינים בתגובה הראשונית, אז אנא אל תחשוב/י זמן רב על כל תשובה |
| --- |

| **מסכים/ה**  **ביותר**  **6** | **מסכים מאוד**  **5** | **מסכים מעט**  **4** | **מעט לא**  **מסכים**  **3** | **מאוד לא**  **מסכים**  **2** | **לא מסכים/ה בכלל**  **1** |
| --- | --- | --- | --- | --- | --- |

| 1 | 2 | 3 | 4 | 5 | 6 | 1. יש כוחות בסיסיים של טוב ורשע בעול |
| --- | --- | --- | --- | --- | --- | --- |
| 1 | 2 | 3 | 4 | 5 | 6 | . כוחות הטוב או כוחות הרשע אחראים למרבית2  האירועים בעולם היום |
| 1 | 2 | 3 | 4 | 5 | 6 | 3. כוחות הטוב והרשע מניעים במקרים רבים התנהגות  אנושית |
| 1 | 2 | 3 | 4 | 5 | 6 | 5. אנשים צריכים להיות מודעים לטוב ולרשע שיש  בעולם היום |
| 1 | 2 | 3 | 4 | 5 | 6 | 8. טוב ורשע הם היבטים של עולם הטבע |

Japanese

| 以下の各文は、あなたが善と悪についてどのように考えているのかについて調べるための ものです。正解や不正解というものはありません。単に、あなたが各文にどのくらい賛成 されるのかを調べようとするものです。あなたがとっさに思い浮かぶ回答を調べたいの で、あまり時間をかけずにお答えください。以下の尺度を使って、あなたがどのくらい賛 成または反対であるかを答えてください。 |
| --- |

| **非常に反対**  **1** | **わりに反対**  **2** | **少し反対**  **3** | **少し賛成**  **4** | **わりに賛成**  **5** | **非常に賛成**  **6** |
| --- | --- | --- | --- | --- | --- |

| 1. この世の根底には善と悪の力が存在している | 1 | 2 | 3 | 4 | 5 | 6 |
| --- | --- | --- | --- | --- | --- | --- |
| 1. 善の力あるいは悪の力のどちらかが、現在の世界で起こ るほとんどの出来事の原因である | 1 | 2 | 3 | 4 | 5 | 6 |
| 1. 善と悪の力はしばしば人の行動の動機となる | 1 | 2 | 3 | 4 | 5 | 6 |
| 1. 人は現在の世界に存在する善や悪に気づく必要がある | 1 | 2 | 3 | 4 | 5 | 6 |
| 1. 善も悪も自然界の一面だ | 1 | 2 | 3 | 4 | 5 | 6 |

Polish

| Poniższe stwierdzenia dotyczą tego w jaki sposób myślisz o **dobru** i **złu**. Nie ma **poprawnych i niepoprawnych odpowiedzi**. Jesteśmy zainteresowani tym w jakim stopniu **zgadzasz się** z każdym z poniższych stwierdzeń. Zależy nam na twoich intuicyjnych odpowiedziach, dlatego nie zastanawiaj się zbyt długo. Proszę zaznacz na poniższej skali jak bardzo **zgadzasz się** lub **nie zgadzasz się** z każdym stwierdzeniem. |
| --- |

| **Zdecydowanie się nie zgadzam**  **1** | **Nie zgadzam się**  **2** | **Raczej się nie zgadzam**  **3** | **Raczej sięzgadzam**  **4** | **Zgadzam się**  **5** | **Zdecydowanie się nie zgadzam**  **6** |
| --- | --- | --- | --- | --- | --- |

| 1. Na świecie działają ukryte siły dobra i zła. | 1 | 2 | 3 | 4 | 5 | 6 |
| --- | --- | --- | --- | --- | --- | --- |
| 1. Za większość wydarzeń w dzisiejszym świecie odpowiadają albo siły dobra albo siły zła. | 1 | 2 | 3 | 4 | 5 | 6 |
| 1. Siły dobra i zła często wpływają na zachowanie ludzi. | 1 | 2 | 3 | 4 | 5 | 6 |
| 1. Ludzie powinni być świadomi dobra i zła obecnych w dzisiejszym świecie. | 1 | 2 | 3 | 4 | 5 | 6 |
| 1. Dobro i zło to dwa aspekty świata natury. | 1 | 2 | 3 | 4 | 5 | 6 |

Portuguese (Brazilian)

| As frases abaixo são sobre o que você pensa sobre o **bem** e o **mal**. Estamos interessados na sua primeira resposta, então, não passe muito tempo pensando sobre cada questão. Para isso, use a escala de resposta abaixo para indicar o quanto você **concorda** ou **discorda** de cada frase. |
| --- |

| **Discordo fortemente**  **1** | **Discordo**  **2** | **Discordo**  **um pouco**  **3** | **Concordo**  **um pouco**  **4** | **Concordo**  **5** | **Concordo fortemente**  **6** |
| --- | --- | --- | --- | --- | --- |

| 1. Neste mundo, existem forças do bem e do mal. | 1 | 2 | 3 | 4 | 5 | 6 |
| --- | --- | --- | --- | --- | --- | --- |
| 1. Forças do bem ou forças do mal são as responsáveis pela maioria dos eventos atuais no mundo. | 1 | 2 | 3 | 4 | 5 | 6 |
| 1. As forças do bem e do mal são, muitas vezes, a causa do comportamento humano. | 1 | 2 | 3 | 4 | 5 | 6 |
| 1. As pessoas precisam estar conscientes do bem e do mal que existe no mundo de hoje. | 1 | 2 | 3 | 4 | 5 | 6 |
| 1. O bem e o mal são aspectos do mundo natural. | 1 | 2 | 3 | 4 | 5 | 6 |

Portuguese (European)

| As afirmações seguintes referem-se a como é que pensa sobre o **bem** e o **mal**. Não há respostas certas ou erradas. Estamos apenas interessados em saber até que ponto concorda com cada uma das afirmações. Estamos interessados nas suas respostas imediatas, por isso, por favor, não demore muito tempo a pensar sobre cada questão. Por favor, use a escala seguinte para indicar até que ponto concorda ou discorda de cada afirmação*.* |
| --- |

| **Discordo fortemente**  **1** | **Discordo moderadamente**  **2** | **Discordo ligeiramente**  **3** | **Concordo ligeiramente**  **4** | **Concordo moderadamente**  **5** | **Concordo fortemente**  **6** |
| --- | --- | --- | --- | --- | --- |

| 1. Neste mundo existem forças do bem e do mal. | 1 | 2 | 3 | 4 | 5 | 6 |
| --- | --- | --- | --- | --- | --- | --- |
| 1. A responsabilidade da maioria dos acontecimentos do mundo de hoje é ou das forças do bem ou das forças do mal. | 1 | 2 | 3 | 4 | 5 | 6 |
| 1. As forças do bem e do mal são, muitas vezes, as causas do comportamento humano. | 1 | 2 | 3 | 4 | 5 | 6 |
| 1. As pessoas precisam estar conscientes do bem e do mal que existe no mundo de hoje. | 1 | 2 | 3 | 4 | 5 | 6 |
| 1. O bem e o mal são aspetos do mundo natural. | 1 | 2 | 3 | 4 | 5 | 6 |

Russian

| Следующие утверждения о том, что вы думаете о **добре** и **зле**. Здесь нет **правильных или неправильных ответов**. Мы только интересуемся тем, насколько вы **согласны** с каждым из следующих утверждений. Нас интересует ваш первый ответ, поэтому не тратьте много времени для обдумывания каждого вопроса. Пожалуйста, используйте следующую шкалу для того, чтобы указать, насколько вы **согласны** или **не согласны** с каждым утверждением. |
| --- |

| **Полностью не согласен**  **1** | **Не согласен**  **2** | **Немного не согласен**  **3** | **Немного согласен**  **4** | **Согласен**  **5** | **Полностью согласен**  **6** |
| --- | --- | --- | --- | --- | --- |

| 1. В этом мире есть основные силы добра и зла. | 1 | 2 | 3 | 4 | 5 | 6 |
| --- | --- | --- | --- | --- | --- | --- |
| 1. Как силы добра, так и силы зла ответственны за большинство событий в современном мире. | 1 | 2 | 3 | 4 | 5 | 6 |
| 1. Силы добра и зла часто мотивируют поведение человека. | 1 | 2 | 3 | 4 | 5 | 6 |
| 1. Людям необходимо быть осведомленными о добре и зле, которые существуют в этом мире. | 1 | 2 | 3 | 4 | 5 | 6 |
| 1. Добро и зло – аспекты мира природы. | 1 | 2 | 3 | 4 | 5 | 6 |

Spanish (Spain)

| Las siguientes afirmaciones se refieren a lo que piensas sobre el **bien** y el **mal**. **No hay respuestas buenas ni malas**. Simplemente estamos interesados en conocer tu grado de **acuerdo** con cada una de las siguiente afirmaciones. Estamos interesados en tus primeras respuestas, de modo que no te tomes mucho tiempo en pensar sobre cada cuestión. Por favor, utiliza la siguiente escala para indicar tu grado de **acuerdo** o **desacuerdo** con cada afirmación. |
| --- |

| **Muy en desacuerdo**  **1** | **Bastante en desacuerdo**  **2** | **Un poco en desacuerdo**  **3** | **Un poco de acuerdo**  **4** | **Bastante de acuerdo**  **5** | **Muy de acuerdo**  **6** |
| --- | --- | --- | --- | --- | --- |

| 1. Hay fuerzas ocultas del bien y el mal en este mundo. | 1 | 2 | 3 | 4 | 5 | 6 |
| --- | --- | --- | --- | --- | --- | --- |
| 1. Tanto las fuerzas del bien como las fuerzas del mal son las responsables de la mayoría de los acontecimientos del mundo hoy en día. | 1 | 2 | 3 | 4 | 5 | 6 |
| 1. Las fuerzas del bien y del mal a menudo motivan el comportamiento humano. | 1 | 2 | 3 | 4 | 5 | 6 |
| 1. La gente necesita ser consciente del bien y del mal que hay en este mundo hoy en día. | 1 | 2 | 3 | 4 | 5 | 6 |
| 1. El bien y el mal son aspectos que forman parte de la naturaleza. | 1 | 2 | 3 | 4 | 5 | 6 |

Spanish (Mexico)

| Las siguientes afirmaciones se refieren a lo que piensas sobre el **bien** y el **mal**. **No hay respuestas buenas ni malas**. Simplemente estamos interesados en conocer tu grado de **acuerdo** con cada una de las siguiente afirmaciones. Estamos interesados en tus primeras respuestas, de modo que no te tomes mucho tiempo en pensar sobre cada cuestión. Por favor, utiliza la siguiente escala para indicar tu grado de **acuerdo** o **desacuerdo** con cada afirmación. |
| --- |

| **Muy en desacuerdo**  **1** | **Bastante en desacuerdo**  **2** | **Un poco en desacuerdo**  **3** | **Un poco de acuerdo**  **4** | **Bastante de acuerdo**  **5** | **Muy de acuerdo**  **6** |
| --- | --- | --- | --- | --- | --- |

| 1. Hay fuerzas ocultas del bien y el mal en este mundo. | 1 | 2 | 3 | 4 | 5 | 6 |
| --- | --- | --- | --- | --- | --- | --- |
| 1. Tanto las fuerzas del bien como las fuerzas del mal son las responsables de la mayoría de los acontecimientos del mundo hoy en día. | 1 | 2 | 3 | 4 | 5 | 6 |
| 1. Las fuerzas del bien y del mal a menudo motivan el comportamiento humano. | 1 | 2 | 3 | 4 | 5 | 6 |
| 1. La gente necesita ser consciente del bien y del mal que hay en este mundo hoy en día. | 1 | 2 | 3 | 4 | 5 | 6 |
| 1. El bien y el mal son aspectos que forman parte de la naturaleza. | 1 | 2 | 3 | 4 | 5 | 6 |

Turkish

| Aşağıdaki ifadeler iyi ve kötü hakkındaki düşüncelerinizle ilgilenmektedir. Doğru ya da yanlış cevap yoktur. Yalnızca her bir açıklamaya ne kadar *katıldığınızla* ilgileniyoruz. Vereceğiniz ilk cevapla ilgileniyoruz, bu nedenle lütfen düşünmek için her bir soruya çok fazla zaman ayırmayınız. Lütfen aşağıdaki ölçeği kullanarak her bir açıklamaya ne kadar *katıldığınızı* ya da *katılmadığınızı* belirtiniz. |
| --- |

| **Kesinlikle katılmıyorum**  **1** | **Kısmen**  **katılmıyorum**  **2** | **Çok az katılmıyorum**  **3** | **Çok az katılıyorum**  **4** | **Kısmen katılıyorum**  **5** | **Kesinlikle katılıyorum**  **6** |
| --- | --- | --- | --- | --- | --- |

| 1. Bu dünyada iyi ve kötünün altında yatan güçler vardır. | 1 | 2 | 3 | 4 | 5 | 6 |
| --- | --- | --- | --- | --- | --- | --- |
| 1. Bugünün dünyasındaki birçok olaydan iyi güçler ya da kötü güçler sorumludur. | 1 | 2 | 3 | 4 | 5 | 6 |
| 1. İyi ya da kötü güçler insan davranışlarını harekete geçirir. | 1 | 2 | 3 | 4 | 5 | 6 |
| 1. İnsanların bugün dünyada olan iyi ve kötünün farkına varması gerekir. | 1 | 2 | 3 | 4 | 5 | 6 |
| 1. İyi ve kötü doğal dünyanın halleridir. | 1 | 2 | 3 | 4 | 5 | 6 |
